# Electrochemical Biosensing of Glucose Based on the Enzymatic Reduction of Glucose

**DOI:** 10.3390/s22197105

**Published:** 2022-09-20

**Authors:** Thomas Soranzo, Awatef Ben Tahar, Ayman Chmayssem, Marc Zelsmann, Pankaj Vadgama, Jean-Luc Lenormand, Phillipe Cinquin, Donald K. Martin, Abdelkader Zebda

**Affiliations:** 1 Univ. Grenoble Alpes, TIMC-IMAG/CNRS/INSERM, UMR 5525, F-38000 Grenoble, France; 2 Univ. Grenoble Alpes, CNRS, CEA-LETI, Grenoble INP, LTM, F-38054 Grenoble, France; 3 School of Engineering and Materials Science, Queen Mary University of London, Mile End Road, London E1 4NS, UK

**Keywords:** electrochemical glucose sensors, aldose reductase, glucose reduction

## Abstract

In this work, the enzyme aldehyde reductase, also known as aldose reductase, was synthesized and cloned from a human gene. Spectrophotometric measurements show that in presence of the nicotinamide adenine dinucleotide phosphate cofactor (NADPH), the aldehyde reductase catalyzed the reduction of glucose to sorbitol. Electrochemical measurements performed on an electrodeposited poly(methylene green)-modified gold electrode showed that in the presence of the enzyme aldehyde reductase, the electrocatalytic oxidation current of NADPH decreased drastically after the addition of glucose. These results demonstrate that aldehyde reductase is an enzyme that allows the construction of an efficient electrochemical glucose biosensor based on glucose reduction.

## 1. Introduction

Glucose measurement is of high importance in a variety of fields, ranging from biomedical applications to ecological approaches. In clinical medicine, diabetes is one of the leading causes of death and disability in the world [1]. Unregulated blood sugar levels in the human body can lead to many life-threatening complications, such as heart disease, stroke, high blood pressure and kidney disease [2,3]. These complications can be delayed and even prevented with careful management of blood glucose (BG) levels [4,5,6]. Thus, it is crucial important to monitor BG levels over time, which requires one to have the analytical means to do so.

The most common method for monitoring of BG levels is regular self-administered capillary blood sampling by the patient and measurement using a biosensor-based glucometer [7,8]. Depending on the detection mode of glucose, many transducers may be used for the design of glucose sensors, such as optical [9], thermal [10] or electrochemical transducers [11]. However, the enzymatic electrochemical biosensors remain the method of choice for glucose detection [12].

Indeed, the most commonly used enzymes for the electrochemical detection of glucose are glucose oxidase (GOx) and glucose dehydrogenase (GDH) [13,14,15]. Both enzymes catalyze glucose oxidation at a fixed potential that is applied at the working electrode. The variation of oxidation current is directly proportional to the concentration of glucose, which leads to the detection of glucose levels [16]. The contributions to that current from the oxidation of some endogenous reducing compound species, such as ascorbate, dopamine and some drugs, can furthermore compromise the selectivity of the glucose biosensors [17,18]. For this reason, different strategies have been developed to minimize this problem, including the use of low reduction potential mediators or by measuring the direct oxidation of the enzyme on the surface of the electrode, also achieved at a low potential [19,20]. These two strategies, respectively, constitute the principles of second and third generation electrochemical glucose biosensors [8]. However, the direct oxidation of the enzyme is a challenging step, and low potential mediators can be complex to use, especially when enzymes are immobilized on the surface of the electrode [21]. Furthermore, the Michaelis constant (K_m_) relative to these enzymes should also be considered. In fact, it is known that these enzymes express a kinetically low intrinsic K_m_ value (around ~1 mM in solution) [16]. Due to this behavior, it is sometimes necessary to dilute samples that present high glucose levels. This is another challenging step that requires one to provide the technical means for allowing the direct analysis of glucose without any dilution.

On the other hand, unlike GOx and GDH, aldehyde reductase (ALDR), also known as aldose reductase, catalyzes the reduction of glucose to sorbitol [22]. ALDR is a barrel-shaped protein consisting of 315 amino acids in a single chain. In the center of the barrel is a binding site for nicotinamide adenine dinucleotide phosphate (NADPH) and another for glucose [23,24]. An acidic hydrogen, from the side-chain of one of the tyrosine amino acids, is positioned close to the aldehyde carbonyl oxygen to serve as the active binding site. Thus, the ALDR enzyme may be used in the construction of an electrochemical glucose biosensor which may allow avoiding of electrochemical interferences and open up the opportunity for undiluted blood glucose analysis due to its higher intrinsic K_m_ property [25].

**Figure 1:**
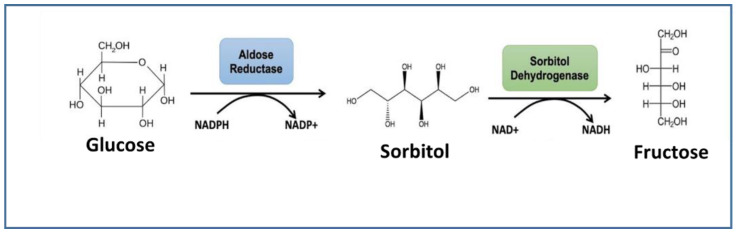
Polyol metabolic pathway.

The work presented here investigates the possibility of performing electrochemical detection of glucose through its reduction by aldehyde reductase. Aldehyde reductase is known to be an NAD(P)H-dependent enzyme that catalyzes the reduction of an aldose to the corresponding sugar alcohol, in particular, D-glucose to sorbitol. Aldose reductase is involved in the polyol pathway, catalyzing the conversion of glucose to sorbitol, which is subsequently converted to fructose by sorbitol dehydrogenase (Figure 1).

Herein, we expressed the enzyme from human origin in *Escherichia coli* (*E. coli*) and characterized its glucose reduction activity by spectrophotometry in the presence of reduced NADPH. Electrochemical investigations confirmed that in the presence of ALDR, the oxidation current of NADPH decreases in the presence of glucose, which is the opposite of classical electrochemical glucose detection methods that typically involve an increase of the oxidation current in the presence of glucose. These results confirm that ALDR can be used as an alternative enzyme to GOx and GDH in order to design glucose biosensors with less interferences complications.

## 2. Experimental

### 2.1. Chemicals

Methylene green (MG) and high viscosity Nafion were purchased from Sigma-Aldrich (Saint Quentin Fallavier, France). Reduced NADPH was purchased from Roche Applied Science (Penzberg, Germany). All reagents were analytical grade, and milli-Q water was used to prepare solutions.

### 2.2. Electrochemical Measurements

BIOLOGIC Instruments’ SP-300 potentiostat interfaced to a PC was used for all electrochemical experiments. A 3-mL-volume three-electrodes electrochemical cell including an Ag/AgCl reference electrode, Pt wire as counter electrode and a plate gold electrode with a geometric area (*A*) of 0.5 cm^2^ as a working electrode was used.

### 2.3. Aldehyde Reductase Production and Assays

The human aldehyde reductase gene (ALDR1) was synthesized and cloned into an expression vector (pD454) by DNA2.0. The protein sequence was fused at its N-Terminal with a hexa-histidine tag and a flexible linker. The plasmid was transformed into BL21 (DE3) *E. coli* bacteria (Novagen, Pretoria, South Africa) and grown in 1 L LB (lysogeny broth) supplemented with 50 µg/mL ampicillin and antifoam 204 (Sigma-Aldrich) in a 5 L baffled shake flask at 16°C for 18 h. Bacteria were then harvested by centrifugation at 3500× *g* for 40 min at 4 °C. After washing with a cold PBS buffer, a 1-g cell pellet was re-suspended in 4 mL of 20 mM Tris, pH 8, 250 mM NaCl and 10 mM imidazole (lysis buffer), supplemented with complete Protease Inhibitor Cocktail, EDTA free (Roche) and DNASE I. Bacteria were passed twice through a French Press (Thermo Electron Corporation, Waltham, MA, USA) at 1500 psi. The cell debris was pelleted by centrifugation at 14,000× *g* for 40 min at 4 °C. A total of 3 mL His-Select™ Nickel Affinity gel (Sigma-Aldrich) was incubated with the bacterial lysate supernatant at 4 °C for 2 h. The beads were washed twice in 20 mM Tris pH 8, 500 mM NaCl and 25 mM imidazole. Proteins were eluted in an elution buffer (20 mM Tris pH8, 500 mM NaCl and 500 mM imidazole) and dialyzed in a dialysis buffer (20 mM Tris pH 8, 10% glycerol and 1 mM DTT). The protein was then subjected to SDS-PAGE and visualized by Coomassie Brilliant Blue (CBB) staining of the gel. The protein concentration was determined by ultraviolet absorption at 280 nm after denaturation in 8 M urea. 

The expressed protein molecular weight was verified by Western blotting after SDS-PAGE migration using a poly-histidine antibody conjugated with horseradish peroxidase (Sigma-Aldrich) diluted at 1:10,000 in TBS-Tween buffer and 5% nonfat milk.

Aldehyde reductase activity was assayed spectrophotometrically by measuring the decrease in the absorption of NADPH at 340 nm after substrate addition using a Jenway Genovan spectrophotometer. Reaction mixtures consisted of 0.1 M potassium phosphate 6.2, 0.2 mM NADPH (Roche), 0.4 mM Li_2_SO_4_, 10 mM Glucose and aldehyde reductase in a total volume of 1 mL. One unit of activity was defined as the activity consuming 1 µmol of NADPH per min at room temperature. An extinction coefficient value of 6.22 mM^−1^·cm^−1^ was used for the NADPH assay.

### 2.4. Electrode Modification

Poly(methylene green) (PMG)-modified gold electrodes were prepared by cyclic voltammetry in the potential range of −1 to + 1 V (vs. Ag/AgCl) at 10 mV·s^−1^ in a phosphate buffer containing 5.0 × 10^−4^ M methylene green [26]. Polymer growth was controlled by the number of deposition cycles. After electropolymerization, the electrodes were thoroughly rinsed with a buffer to remove any remaining monomeric MG. The immobilization of the enzyme ALDR and its cofactor NADPH was achieved as already described in the literature [27]. Briefly, 0.71 mg of ALDR were mixed to 3 mg of NADPH in 10 µL of Nafion-modified tetrabutylammonium fluoride (TBAF). The mixture was pipetted onto the gold electrodes surface and dried for at least 2 h at an ambient temperature. 

## 3. Results and Discussion

In this part, the aldehyde reductase (ALDR) enzyme was characterized after its biosynthesis by two means: Coomassie Brilliant Blue staining technique and by Western-blotting analysis. The electrodeposition of poly(methylene green) (PMG) on the surface of gold-electrodes was also investigated. Then, the electrocatalytic activity of PMG was studied in presence of nicotinamide adenine dinucleotide phosphate (NADPH) to determine the potential of oxidation of NADPH on the modified gold electrodes. In the last part, we investigated the ability of ALDR to catalyze the reduction of glucose to sorbitol and the oxidation of NADPH to NADP, aiming to develop a glucose biosensor based on enzymatic reduction activity of ALDR in the presence of glucose.

### 3.1. Characterization of Aldehyde Reductase 

**Figure 2:**
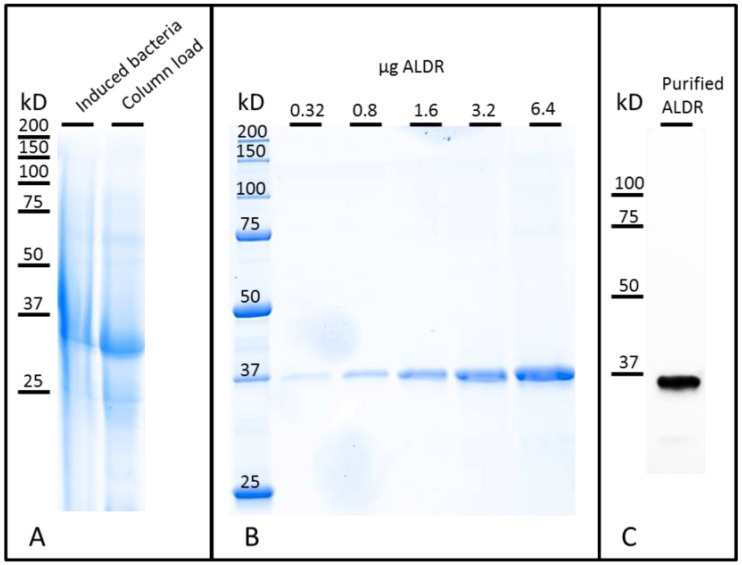
ALDR electrophoresis analysis. (**A**) Total protein before purification (CBB). (**B**) Observation of different quantities of purified ALDR (CBB). (**C**) Visualization of the purified ALDR using western blotting.

To produce the aldehyde reductase (ALDR) recombinant protein, the complete human protein sequence was fused to an N-terminal hexa-histidine affinity tag. Figure 2 shows the nickel affinity chromatography purification of the his-tagged recombinant fusion protein after *E. coli* expression. The purity of the ALDR was confirmed by Coomassie Brilliant Blue staining (Figure 2B) and by Western-blotting using anti-his tag antibodies (Figure 2C). The purity was evaluated by SDS-PAGE as >99% with 1.6 µg of the enzyme (Figure 2B). The estimated concentration was 3.2 mg×mL^−1^ after dialysis using absorbance at 280 nm. For this, the protein was denaturated in 8 M urea, and the concentration was calculated using an extinction coefficient of 49,390 M^−1^×cm^−1^ (Protparam [28]). The calculated molecular weight based on the amino acid sequence for the his-tagged aldehyde reductase was 37.4 kD. Western-blotting analysis (Figure 2C) confirmed the expected molecular weight, showing the expression of a full-length ALDR.

The enzymatic activity was assayed spectrophotometrically by monitoring NADPH oxidation over time at 340 nm. Specific activity based on the difference in NADPH concentration after 3 min of the reaction was 12 U·mg^−1^. The preparation protocol used therefore enabled the expression of highly active aldehyde reductase at a high yield. The estimated yield of ALDR was approximately 120 mg·L^−1^ of bacterial culture. 

### 3.2. Electrodeposition of Poly(Methylene Green)

The use of poly(methylene green) (PMG) has widely emerged in the last years [25,29,30]. It is basically used because of its physical properties as conductive polymer, for its stability and for its advantageous electrocatalytic properties for NADH and NADPH [31]. PMG can easily be deposed by electropolymerization on the surface of electrodes, which represents a very outstanding feature. The latest allows the more precise control of the film thickness and its growth [32]. 

**Figure 3:**
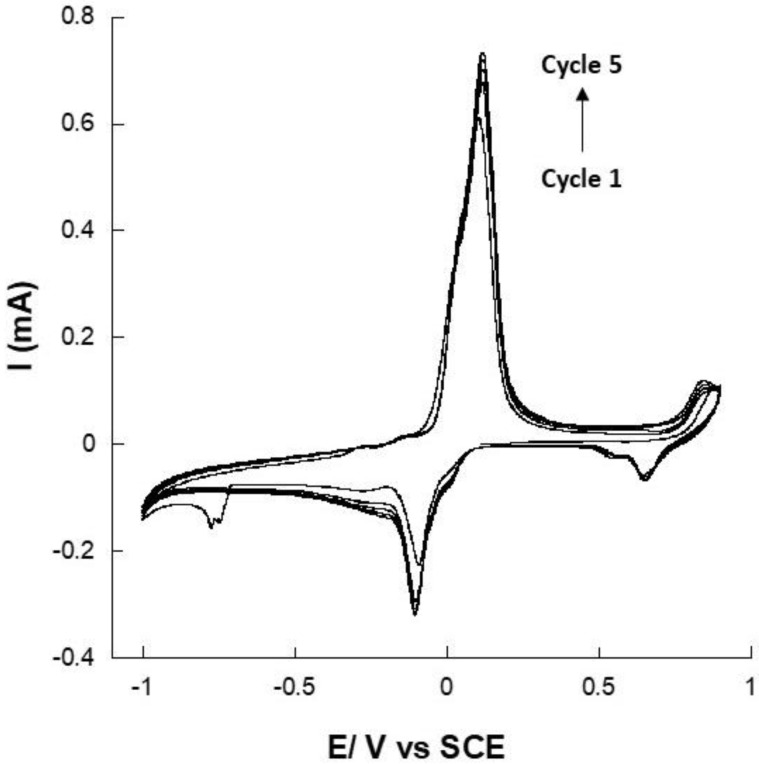
Consecutive cyclic voltammograms at 10 mV·s^−1^ of methylene green (MG) electropolymerization on a gold electrode in pH 7 PBS containing 5.0 × 10^−4^ M MG (5 cycles).

Representative cyclic voltammograms at 10 mV·s^−1^ for electropolymerization of poly(Methylene Green) (PMG) on the electrode surface are presented in Figure 3. The oxidative peak of MG was observed at +0.1 V in the first cycle and at +0.11 V in the 5th cycle, vs. Ag/AgCl. In the same way, the reduction peak shifted to the negative’s values, and its intensity increased in absolute terms. These shifts of the oxidative and reduction peaks and the increasing of the current intensity indicate that an oxidative polymerization was taking place and that more electroactive species were being deposited on the surface of the electrode [26,33,34]. 

Regarding the polymerization process, Yevgenia and his co-authors have already reported that the polymerization of methylene green occurs by forming a cation-radical, which dimerizes to yield an –NH– bridge between the two monomers [32]. Obviously, the PMG membrane’s thickness is dependent on the number of cycles done by cyclic voltammetry. The thickness was much higher as the number of cycles increased. However, mechanical properties and stability of the formed film were gradually affected by the cycles number as well as the film conductivity. In our previous work, we showed that electropolymerization of MG could increase the current density up to 100 times due to the geometric surface area increasing [25]. However, the optimum number of cycles was obtained at 10 scans, which were conducted to obtain a very thin film. We already demonstrated that large polymer quantities on the surface of electrode may drastically decrease the surface conductivity and thus limit the electron exchange. Here, the Faradic current increased until cycle 5. Thereafter, the capacitive current started to increase, and the Faradic current was stabilizing. Thus, an optimized cycles number of 5 was fixed for the electropolymerization on a gold electrode of MG.

### 3.3. NADPH Electrocatalysis with PMG Modified Gold Electrode

**Figure 4:**
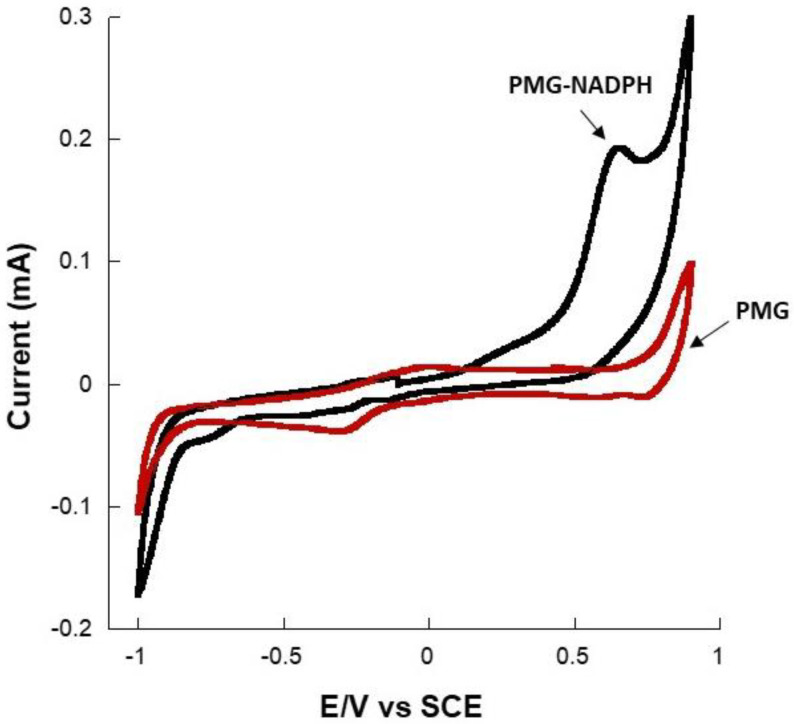
Representative cyclic voltammograms at 2 mV·s^−1^ showing the oxidation of NADPH on a PMG-modified gold electrode in pH 7 PBS.

The modified gold-electrodes were tested for their ability to catalyze the oxidation of NADPH. Indeed, PMG has been previously used as an NADH and NADPH regeneration matrix [35,36]. Here, cyclic voltammetry (CV) experiments were undertaken with PMG-modified gold electrodes at 2 mV·s^−1^ to determine the potential at which the electro-oxidation of NADPH takes place. As expected, the CV of a PMG modified electrode in the presence of NADPH presented an oxidation peak at +0.62 V (Figure 4), in agreement with results reported on the electro-oxidation of NADPH and in agreement with reported studies on the capacity of PMG, like other azine dyes, to regenerate NADPH [36,37,38]. 

### 3.4. Catalytic Reduction of Glucose

**Figure 5:**
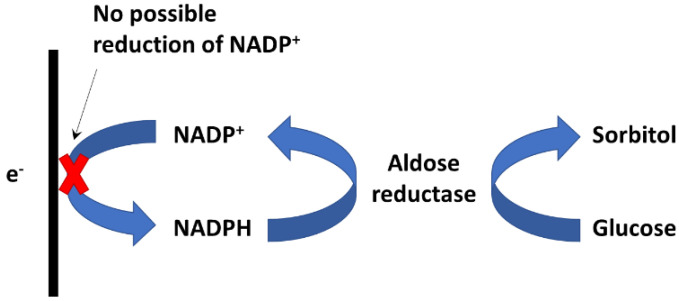
Schema of catalytic reduction of glucose to sorbitol by the enzyme aldehyde reductase (ALDR). Left, as is known, the NADP^+^ reduction is not possible on the kind of electrode.

In the presence of glucose and NADPH, ALDR is able to catalyze the reduction of glucose to sorbitol and the oxidation of NADPH to NADP (Figure 5). Thus, in this case, we should observe a decrease in NADPH concentration.

**Figure 6:**
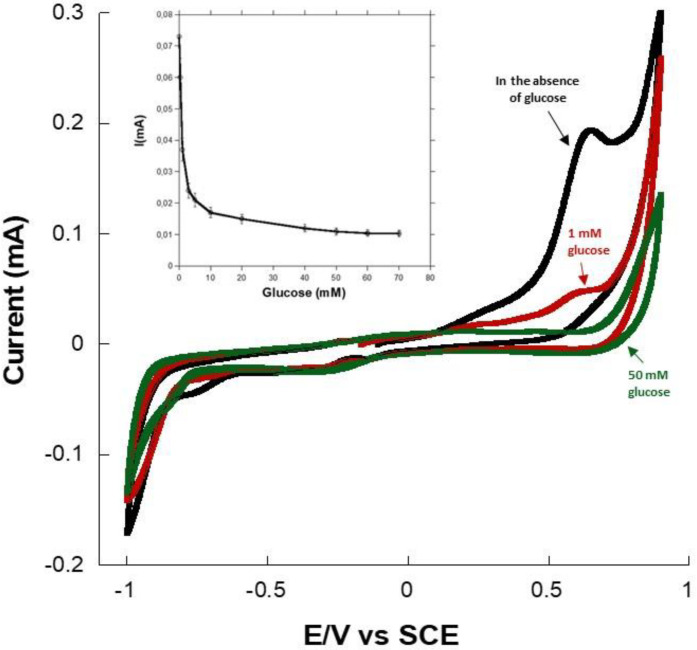
Cyclic voltammograms of the gold–PMG–ALDR–NADPH electrode at 2 mV·s^−1^ examined in pH 7 PBS in the absence and the presence of 1- and 50-mM glucose. Inset, oxidation current at +0.62 V as a function of glucose concentration (3 repetitions).

Figure 6 shows the CV of a PMG-modified gold electrode containing ALDR and NADPH, in the absence and in the presence of glucose (at 2 mV·s^−1^). Herein, we observed that in the absence of glucose, the CV exhibited an oxidation peak at +0.62 V vs. Ag/AgCl, corresponding to the catalytic oxidation of NADPH to NADP. However, when glucose was added to the buffer solution, we observed a decrease in the oxidation intensity. Moreover, when the concentration of glucose increased to 50 mM, the oxidation peak of NADPH completely disappeared. Indeed, the NADPH oxidation peak on the CV curves (Figure 5 inset) decreased as the concentration of glucose increased in the solution. At a high glucose concentration, the NADPH oxidation peak completely disappeared. The presence of glucose drove the enzymatic oxidation of NADPH to NADH by ALDR, which involved a decrease of the NADPH concentration on the surface of the electrode and, consequently, a reduction of the NADPH oxidation current [38]. When the concentration of glucose is high, all the NADPH molecules are oxidized to NADP by ALDR, and no NADPH oxidation current can thus be observed, since there is no NADPH on the surface of the modified electrode. Inset Figure 5 shows the evolution of the NADPH CV oxidation current at 620 mV with the glucose concentration, and we can see that the oxidation current decreased with the decrease of the glucose concentration. This decrease exhibited a linear behavior until 3 mM, and after that, the biosensor deviated from linearity and saturated thereafter.

To verify the response mechanism, the experiment was repeated in the absence of ALDR (Figures S1 and S2). In this case, glucose had no effect on the NADPH oxidation current, which confirmed the enzymatic consumption of NADPH in the presence of glucose. Thus, the catalytic reduction of glucose to sorbitol by the enzyme aldehyde reductase was confirmed, which validated the hypothesis we proposed for the catalytic mechanism in Figure 5.

These results clearly show that unlike glucose dehydrogenase-based glucose biosensors, ALDR-based glucose detection is based on a catalytic reduction that provides an electrochemical detection of glucose from the measurement of a decrease in the intensity of the NADPH oxidation current. Conversely to the first generation of glucose biosensors that rely on the use of the natural oxygen as a co-substrate and the generation and detection of hydrogen peroxide (H_2_O_2_) [16], the enzyme aldehyde reductase does not involve, in its catalytic mechanism, any dependence on the oxygen, and as a result, it does not address any oxygen limitation [25]. Whilst the peak potential is similar to that for H_2_O_2_ as required in GOx first generation glucose biosensors [39,40], the present electrode would not be affected by a variable background pO_2_ nor non-electrochemical external H_2_O_2_ degradation due to a sample catalase. Moreover, electrochemical glucose biosensors often suffer from the presence of interferents, such as ascorbic acid, that are exhibited on oxidation currents, and in our case, monitoring glucose reduction may prevent the interferents’ effects on the biosensor response. These characteristics open up the opportunity to the ALDR-based glucose detection to be a new alternative for electrochemical glucose sensing.

## 4. Conclusions

Aldehyde reductase, recombinantly expressed from human origin, has shown the capacity to catalyze, in the presence of NADPH, the reduction of glucose to sorbitol. Electrochemical measurements showed that, in the presence of aldose reductase and glucose, the NADPH oxidation current on a PMG-modified gold electrode decreased significantly. This study showed that NADPH can be reproducibly monitored without electrode passivation using PMG and that the problem of H_2_O_2_ instability can be avoided along with the long-term toxic effect of H_2_O_2_ on GOx. Finally, the future challenge for this system is the development of an electrode that can electrocatalyze the reduction of NADPH, which would pave the way for the development of glucose sensors based on the measurement of the glucose reduction current at a low potential. Furthermore, the intrinsic high K_m_ of ALDR opens up the opportunity for undiluted blood glucose analysis with reduced dependence on electronic curve fitting.

## Data Availability

The data that support the findings of this study are available from the corresponding author upon reasonable request.

## Supplementary Materials

The following supporting information can be downloaded at: https://www.mdpi.com/article/10.3390/s22197105/s1, Figure S1: Chronoamperometric responses (at +0.25 V) of PMG-NADPH-Nafion modified gold electrode and PMG-NADPH-ALDR-Nafion modified gold electrode in pH 7 PBS in presence of glucose (50 mM); Figure S2: Representative cyclic voltammogram at 2 mV·s^−1^ of NADPH oxidation on PMG-NADPH-Nafion modified gold electrode in pH 7 PBS in presence of glucose (50 mM).
