# Supplementary material for: Electrochemical Biosensing of Glucose Based on the Enzymatic Reduction of Glucose
Source: Sensors (Basel). 2022 Sep 20;22(19):7105. doi: 10.3390/s22197105 (PMC9572614; doi:10.3390/s22197105)
Supplement: Supplementary file 1 [file sensors-22-07105-s001.zip › sensors-1888397-supplementary.pdf]

sensors

MDPI

Supplementary information

Electrochemical Biosensing Of Glucose Based on Enzymatic Reduction Of Glucose

Thomas Soranzo, [1] Awatef Ben Tahar, [1] Ayman Chmayssem, [1] Marc Zelsmann, [2] Pankaj Vadgama, [3] Jean-Luc Lenormand, [1] Phillipe Cinquin, [1] Donald K. Martin, [1] Abdelkader Zebda [1*]

[1] Univ. Grenoble Alpes, TIMC-IMAG/CNRS/INSERM, UMR 5525, F-38000 Grenoble, France
[2] Univ. Grenoble Alpes, CNRS, CEA-LETI, Grenoble INP, LTM, F-38054 Grenoble, France
[3] Queen Mary University of London, Mile End Road, E14N, London, United Kingdom
* Correspondence: abdelkader.zebda@univ-grenoble-alpes.fr

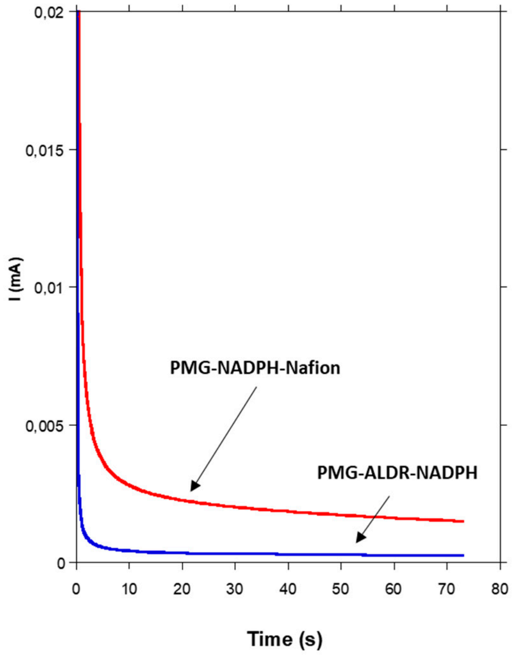

Figure S1. Chronoamperometric responses (at + 0,25 V) of (red) PMG-NADPH-Nafion modified gold electrode and (blue) PMG-NADPH-ALDR-Nafion modified gold electrode in pH 7 PBS in presence of glucose (50 mM).

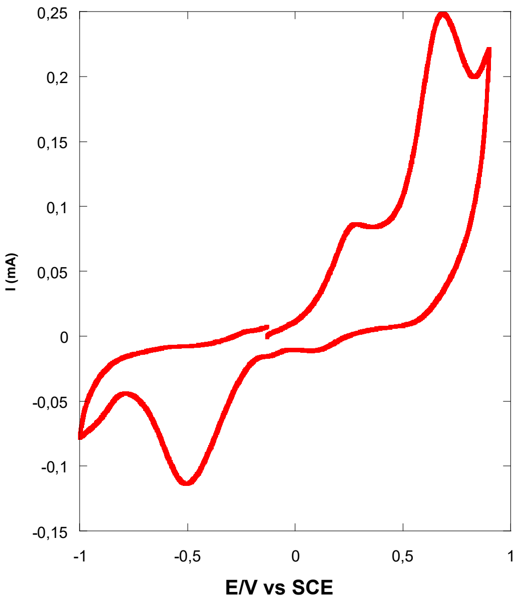

Figure S2. Representative cyclic voltammogram at 2 mV.s^{-1} of NADPH oxidation on PMG-NADPH-Nafion modified gold electrode in pH 7 PBS in presence of glucose (50 mM).
